# TCD hemodynamics findings in the subacute phase of anterior circulation stroke patients treated with mechanical thrombectomy

**DOI:** 10.1515/med-2022-0464

**Published:** 2022-03-28

**Authors:** Tingting Wu, Zongjie Shi, Bo Chen, Yu Geng, Jie Pan

**Affiliations:** Department of Neurology, Zhejiang Provincial People’s Hospital (Affiliated People’s Hospital, Hangzhou Medical College), Hangzhou, 310014, Zhejiang China; Medical College of Soochow University, Suzhou, 215123, China; Department of Neurology, Zhejiang Provincial People’s Hospital (Affiliated People’s Hospital, Hangzhou Medical College), No. 158 Shangtang Road, Hangzhou, 310014, Zhejiang China

**Keywords:** transcranial Doppler ultrasound, thrombectomy, infarction, anterior cerebral artery, stroke

## Abstract

Despite mechanical thrombectomy, the prognosis for many patients with anterior circulation ischemic stroke (ACIS) remains poor. This retrospective study reports consecutive mechanical thrombectomy procedures for ACIS at our hospital over 4 years. Hemodynamics were explored using transcranial Doppler ultrasound. The functional outcome was assessed using the modified Rankin scale. A total of 121 eligible cases were included: 61 (50.4%) exhibited good outcomes (modified Rankin scale score ≤2) by day 90. The logistic regression analysis showed that ipsilateral middle cerebral artery (iMCA) systolic blood flow (SBF) (OR = 0.983, 95% CI: 0.969–0.997, *P* = 0.014), preoperative National Institutes of Health Stroke Scale (NIHSS)score (OR = 1.160, 95% CI: 1.067–1.261, *P* < 0.001), intracranial hemorrhage after therapy (OR = 19.514, 95% CI: 4.364–87.265, *P* < 0.001), and Alberta Stroke Program Early Computed Tomography Score (OR = 0.639, 95% CI: 0.416–0.981, *P* = 0.040) were independently associated with prognosis. The iMCA SBF and preoperative NIHSS score were significantly predictive of a good outcome in the receiver operating characteristic analysis. In conclusion, elevated iMCA SBF might be a prognostic indicator of a good 90-day outcome following endovascular treatment in ACIS patients treated with mechanical thrombectomy, but large prospective studies are mandatory to validate the findings of our study.

## 1 Introduction

Ischemic stroke of the anterior circulation accounts for 80–87% of all strokes worldwide, with a global incidence of 2–3 cases per 1,000 people per year [1–3]. Such strokes are the fifth leading cause of early mortality worldwide [4]. The etiology of ischemic stroke is characterized by obstructive thrombotic or embolic events decreasing blood flow to the brain.

Significant advancements in acute stroke care have been made in the last decade, but outcomes are still associated with etiological factors and rapid intervention. Multiple stroke trials demonstrated that endovascular thrombectomy within the initial 6 h from the onset in patients with large vessel occlusion of the anterior circulation resulted in superior outcomes, leading to the adoption of criteria like diffusion-weighted imaging or computed tomography perfusion assessment with clinical mismatch in the triage of wake-up and late presenting strokes undergoing neurointervention with trevo (DAWN) and endovascular therapy following imaging evaluation for ischemic stroke 3 (DEFUSE 3) [5,6]. Mechanical thrombectomy in the acute period (initial 6 h) is the standard treatment for acute ischemic stroke patients with large cerebral vessel occlusion, reducing the magnitude of the final infarct core and thus improving clinical outcomes [7,8].

Despite these advances, death or functional impairment is still observed after thrombectomy, indicated by a modified Rankin scale (mRS) score >2 (moderate-to-severe disability) [5–8]. For example, Nogueira et al. [5] showed that the rate of functional independence after thrombectomy was 49%. Contributing factors include the occurrence of intracranial hemorrhage (ICH), secondary thrombus, and cerebral vasospasm, which are not readily visible with current clinical approaches [9–11].

Hemodynamic findings during an ischemic stroke are closely associated with complications and prognosis [12]. Transcranial Doppler ultrasound (TCD) is a noninvasive tool that allows real-time evaluation of cerebrovascular hemodynamics, including blood flow and vascular functional status [13]. TCD can effectively demonstrate spectral flow waveforms, blood flow direction, velocities, and intensity in the intracerebral vessels, adding further physiological information about both sides of the anterior cerebral artery (ACA) or posterior cerebral artery (PCA) in an acute middle cerebral artery (MCA) or internal carotid artery (ICA) occlusion [14]. Still, further research is needed to confirm the prognostic significance of TCD hemodynamic findings in anterior circulation ischemic stroke (ACIS).

Therefore, this study examined the association between acute hemodynamic findings on TCD and patient outcomes following a mechanical thrombectomy.

## 2 Methods

### 2.1 Study design and patient selection

A retrospective analysis was performed on consecutive patients treated with mechanical thrombectomy for ischemic stroke and acute large vessel occlusion of the anterior circulation (i.e., intracranial ICA or MCA main stem occlusion) at the Department of Neurology of Zhejiang Provincial People’s Hospital of China between June 1, 2016, and August 31, 2020. The approval for the use of patient data from the institutional electronic stroke database and exemption from prospective informed consent was provided by the local Research Ethics Committee. All identifiable patient information was removed to ensure patient confidentiality.

The inclusion criteria were (1) adults (≥18 years of age), (2) pre-stroke mRS score of 0–1 [15], (3) causative occlusion of the ICA or proximal MCA (M1, i.e., horizontal segment) by digital subtraction angiography (DSA), (4) received intravenous recombinant tissue plasminogen activator within 4.5 h of onset [2], (5) National Institutes of Health Stroke Scale (NIHSS) score ≥6 [16], (6) Alberta Stroke Program Early computed tomography (CT) score (ASPECTS) ≥ 6 [17], and (7) treated within 6 h of symptom onset.

The exclusion criteria were (1) recanalization was incomplete (defined as Thrombolysis in Cerebral Infarction (TICI) grades 0–2a), (2) the stenosis of the ipsilateral ICA, MCA, or PCA was more than 50% on TCD, CT angiography, or DSA, or (3) no TCD data on record or no apparent temporal window for TCD examination were not included.

### 2.2 Mechanical thrombectomy procedure

Mechanical thrombectomy was performed according to the standard of care using routine stent retrievers or clot aspiration systems. Procedures were performed by four experienced interventional radiologists (>20 procedures) working in teams of two. All procedures were performed under general anesthesia or conscious sedation, as determined by the attending anesthesiologist.

### 2.3 TCD ultrasound examination

**Figure 1:**
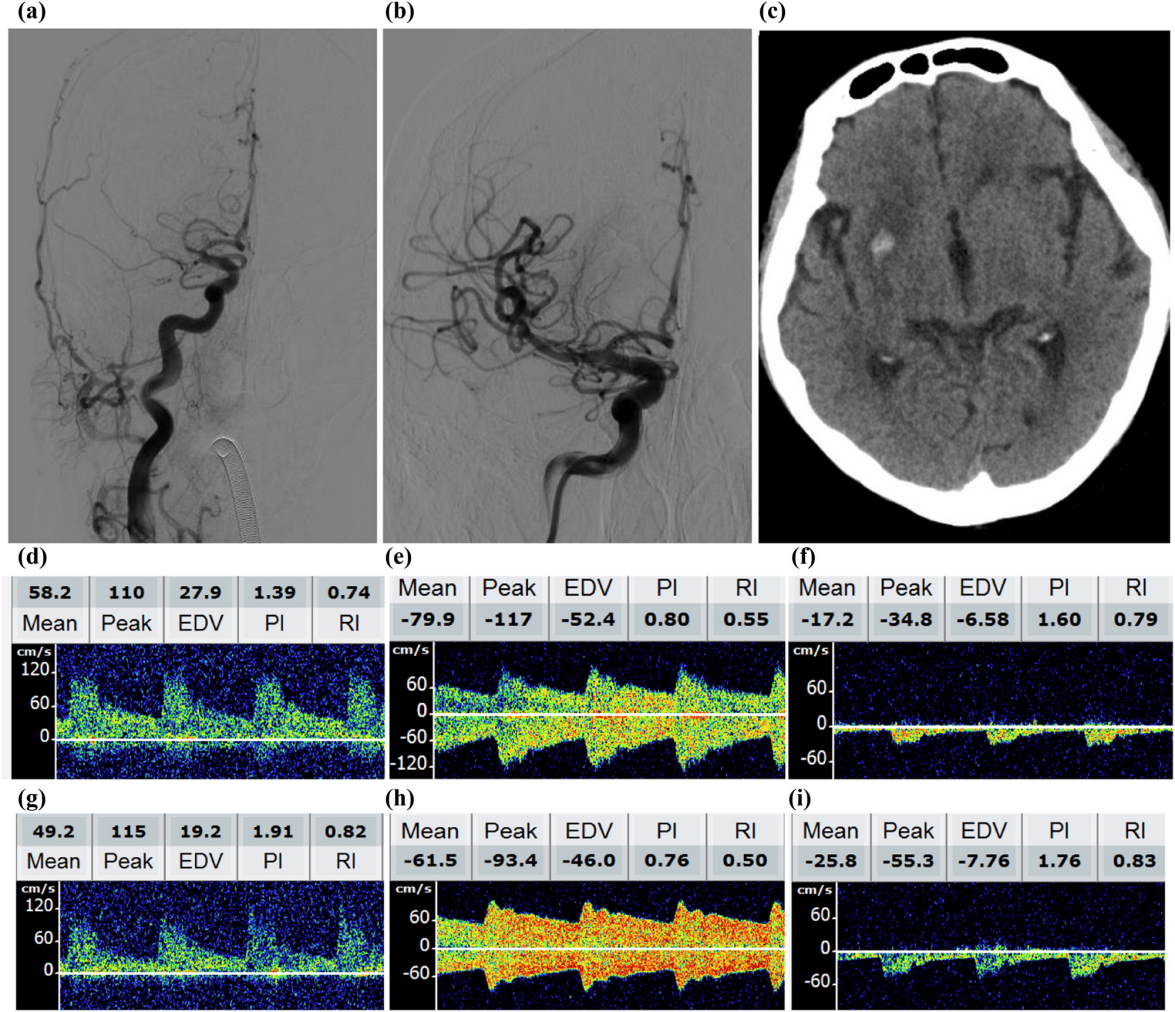
Representative images of a 78-year-old patient with MCA occlusion who underwent mechanical thrombectomy and achieved a 90-day mRS score of 0. (a) Pre-surgical DSA confirmed proximal MCA occlusion (right). (b) DSA after successful recanalization with a solitaire stent retriever shows MCA reperfusion. (c) CT at 24 h after mechanical thrombectomy shows nonsymptomatic ICH. Postinterventional transcranial duplex sonography 7 days after recanalization shows the ipsilateral (i) MCA (d), contralateral (c) MCA (g), a much higher c ACA SBF, and cACA RI (h) compared with iACA (e); iPCA SBF velocity index and much lower iPCA SBF (e) compared with the contralateral PCA (i).

All patients underwent a post-procedure TCD assessment. TCD was performed using handheld ultrasound transducers (2 MHz PW or 4 MHz PW/CW; Natus Neurology Inc., Middleton, WI, USA) by trained sonographers between 10 and 14 days after mechanical thrombectomy procedures or before discharge. Notably, the MCA is typically located 50–55 mm deep and recognizable by blood flow toward the probe, while the ACA is typically located 60–70 mm deep and recognizable by its characteristic blood flow away from the probe [13,18]. As the probe is positioned toward the posterior and inferior regions, the PCA is identified 55–70 mm deep with the flow directed away from the probe (P2 segment). Values for blood flow velocities, including systolic blood flow (SBF), diastolic blood flow (DBF), mean blood flow (MBF), pulsatility index, and resistance index (RI), were analyzed in the vessels of the treated ipsilateral and contralateral sides. Figure 1 presents images of a patient with acute MCA occlusion who underwent mechanical thrombectomy and achieved a 90-day mRS score of 0. TCD examination was not performed if a patient had critical symptoms after thrombectomy and was transferred to the intensive care unit.

### 2.4 Clinical outcomes

All patients underwent cerebral CT imaging 24 h after thrombectomy or in the event of clinical deterioration. ICH was evaluated for 14 days post-procedure or immediately before discharge. Imaging data were reviewed for ICH by trained radiologists blind to TCD data. ICH was defined using the Heidelberg Bleeding Classification [15], and symptomatic ICH was defined as worsening symptoms in a patient who had an increase of >4 points in the NIHSS score. Clinical neurological examinations were performed 87–93 days after the procedure. Clinical outcomes were assessed using the mRS score [19], with an mRS score of ≤2 defined as a good outcome.

### 2.5 Data collection

All data were collected retrospectively from medical records found in the institutional electronic stroke database, which compiles patient demographics, history (including cerebrovascular risk factors, previous vascular events, and medication use), stroke-related diagnosis and therapy, stroke complications, and outcomes.

### 2.6 Statistical analysis

Statistical analyses were performed using SPSS 23.0 (IBM, Armonk, NY, USA). Continuous variables were presented as mean ± standard deviation/median (interquartile range) according to the Kolmogorov–Smirnov test and analyzed using Student’s *t*-test/Mann–Whitney U test. Categorical variables were presented as percentages and analyzed using the chi-square test. Variables that were statistically different between groups were analyzed by multivariable logistic regression analysis. Receiver operating characteristic (ROC) curve analysis was used to determine the sensitivity and specificity of the new assessment. *P*-values less than 0.05 (*P* < 0.05) were considered statistically significant.

## 3 Results

### 3.1 Patient demographics and clinical characteristics

Of 298 patients with acute ischemic stroke who underwent mechanical thrombectomy for an ICA or MCA main stem occlusion during the study period, 177 (59.4%) were excluded: 5 (2.8%) were excluded due to incomplete vessel recanalization (TICI 0–2a), 43 (24.3%) due to >50% additional carotid artery or MCA stenosis or vessel occlusions other than in the one receiving the intervention, 101 (57.1%) due to lack of TCD data, and 28 (15.8%) due to no temporal window for TCD.

**Figure 2:**
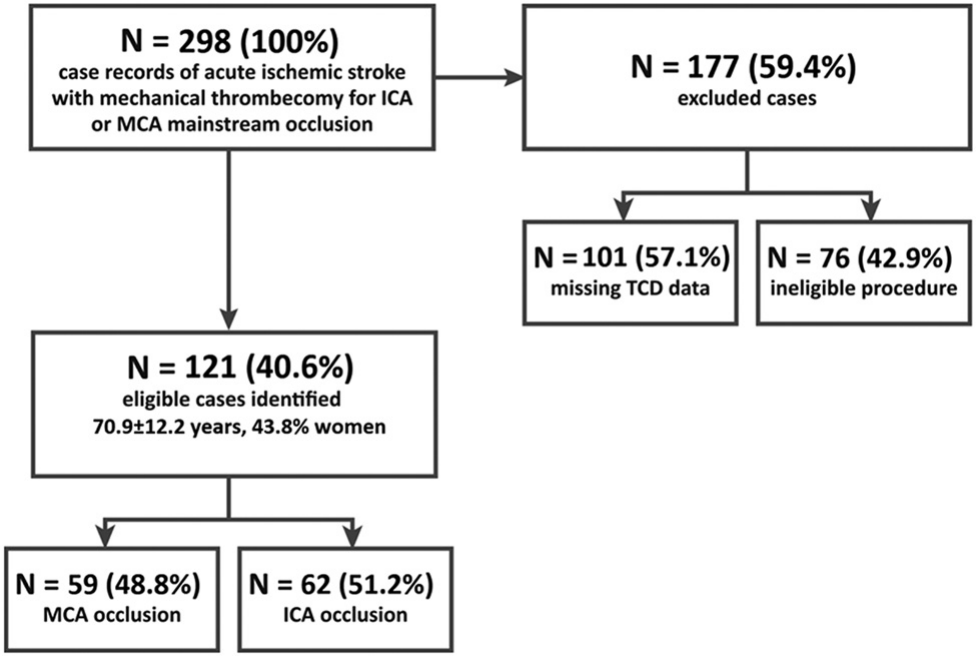
Flowchart schematic of included and excludes cases in this retrospective study.

Therefore, 121 eligible patients were identified (70.9 ± 12.2 years, 43.8% women), of which 59 (48.8%) had an MCA occlusion and 62 (51.2%) had an ICA occlusion (Figure 2). Mechanical thrombectomy was performed primarily with stent retrievers (95.0%).

Demographics, medical history, acute stroke therapy, and clinical/diagnostic findings for the 121 included patients are summarized in Table 1. The patients were stratified according to an mRS score ≤2 (*n* = 61, 50.4%). Patients with good outcomes exhibited significantly lower age of onset (*t*-test, *P* = 0.010), lower NIHSS score at onset (*t*-test, *P* < 0.001) and ASPECTS (*t*-test, *P* = 0.038). Patients with poor outcomes had a higher rate of post-procedural ICH (chi-square test, *P* < 0.001).

**Table 1:** Demographics, medical history, acute stroke therapy, and clinical/diagnostic findings in patients with ischemic stroke successfully treated for anterior circulation vessel occlusion by mechanical thrombectomy

Characteristics	All Patients (*n* = 121)	Day 90 mRS ≤2	Day 90 mRS >2	*P*
		(*n* = 61)	(*n* = 60)	
Age (years)	70.93 ± 12.16	68.13 ± 12.46	73.77 ± 11.24	0.010
Gender (female)	53 (43.8%)	28 (45.9%)	25 (41.7%)	0.775
Arterial hypertension	90 (74.4%)	45 (73.8%)	45 (75.0%)	0.957
Diabetes mellitus	17 (14.0%)	7 (11.5%)	10 (16.7%)	0.575
Nicotine use	22 (18.2%)	12 (19.7%)	10 (16.7%)	0.847
Atrial fibrillation	86 (71.1%)	39 (63.9%)	47 (78.3%)	0.122
Preoperative NIHSS score	19.07 ± 6.57	17.0 ± 5.74	21.2 ± 6.7	<0.001
Onset to recanalization time (min)	435.56 ± 209.34	415.30 ± 170.82	459.33 ± 246.68	0.281
Systolic BP (mmHg)	151 ± 25.17	148.58 ± 26.02	153.30 ± 24.26	0.299
Diastolic BP (mmHg)	86.66 ± 16.41	84.4 ± 16.10	88.95 ± 16.54	0.131
Blood glucose (mmol/L)	7.47 ± 2.29	7.36 ± 1.84	7.58 ± 2.67	0.596
Platelet count (×10^9^/L)	174.2 ± 62.35	179.44 ± 55.50	168.78 ± 68.68	0.350
INR	1.05 ± 0.16	1.05 ± 0.17	1.08 ± 0.17	0.529
ASPECTS	9 (8, 10)	9 (8, 10)	9 (8, 9)	0.040
Acute Stroke Therapy				0.160
Thrombectomy	73 (59.5%)	32 (52.5%)	40 (66.7%)	
Thrombectomy with thrombolytic therapy	49 (40.5%)	29 (47.5%)	20 (33.3%)	
Lesion Location				0.071
ICA	40 (33.06%)	15 (24.6%)	25 (41.7%)	
MCA	81 (66.9%)	46 (75.4%)	35 (58.3%)	
ICH after therapy	27 (22.3%)	3 (4.9%)	24 (40.0%)	<0.001
Duration between surgery and the date of assessment	90 (88, 90.5)	90 (88, 90.5)	90 (88.25, 90.75)	0.797

BP: blood pressure; ICH: intracerebral hemorrhage; INR: International normalized ratio.

### 3.2 TCD iMCA velocity is predictive of good 90-day outcome

TCD ultrasonography showed that patients with good outcomes exhibited significantly higher iMCA SBF (*P* < 0.001), iMCA DBF (*P* < 0.001), and iMCA MBF (*P* < 0.001) (Table 2).

**Table 2:** Postinterventional transcranial duplex sonography findings in patients with different 90-day mRS scores treated by mechanical thrombectomy

Variables	All Patients (*n* = 121)		Day 90 mRS ≤2 (*n* = 61)		Day 90 mRS >2 (*n* = 60)		*t*-test *P*
	Mean ± SD	95% CI	Mean ± SD	95% CI	Mean ± SD	95% CI	
iMCA SBF	86.73 ± 21.90	82.09–91.39	97.68 ± 17.81	92.13–103.23	74.31 ± 19.48	68.53–80.09	<0.001
iMCA DBF	33.90 ± 11.89	331.38–36.42	38.30 ± 10.43	35.05–41.55	28.92 ± 11.59	25.48–32.36	<0.001
iMCA MBF	51.51 ± 14.14	48.51–54.51	58.09 ± 11.52	54.50–61.68	44.05 ± 13.20	40.13–47.97	<0.001

SD: standard deviation; CI: confidence interval.

The logistic regression analysis showed that iMCA SBF (OR = 0.983, 95% CI: 0.969–0.997, *P* = 0.014), preoperative NIHSS score (OR = 1.160, 95% CI: 1.067–1.261, *P* < 0.001), ICH after therapy (OR = 19.514, 95% CI: 4.364–87.265, *P* < 0.001), and ASPECTS (OR = 0.639, 95% CI: 0.416–0.981, *P* = 0.040) were independently associated with prognosis (Table 3).

**Table 3:** Multivariable analysis of the factors associated with a good prognosis

Variables	OR	95%	*P*
iMCA systolic blood flow	0.983	0.969–0.997	0.014
Preoperative NIHSS score	1.160	1.067–1.261	<0.001
ICH after therapy	19.514	4.364–87.265	<0.001
ASPECTS	0.639	0.416–0.981	0.040

**Figure 3:**
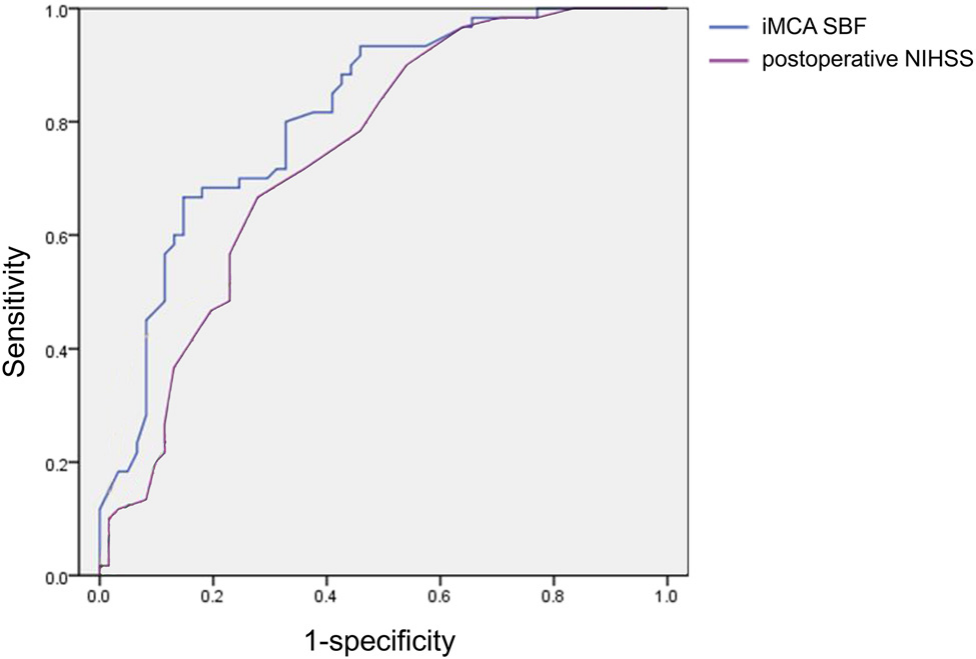
Predictive ability of the ipsilateral middle cerebral artery (iMCA) systolic blood flow (SBF) and preoperative NIHSS score for a good outcome (90-day mRS score ≤2).

Of these hemodynamic parameters, ROC analysis further revealed that iMCA SBF (area under the ROC curve (AUC) = 0.819, 95% CI: 0.716–0.896, *P* < 0.001) was significantly predictable for a good outcome (90-day mRS ≤ 2). Optimal sensitivity (Se = 70.27%) and specificity (Sp = 88.10%) were achieved with a cutoff value of 82 (Figure 3). Preoperative NIHSS score (AUC = 0.742, 95% CI: 0.653–0.830, *P* < 0.001) was significantly predictive for a good outcome (90-day mRS ≤ 2). Optimal sensitivity (Se = 66.70%) and specificity (Sp = 72.10%) were achieved with a cutoff value of 19.5.

## 4 Discussion

This study provided novel insights into the correlative relationship between hemodynamic findings apparent in TCD ultrasonography and functional outcomes, based on the 90-day mRS score, in patients undergoing mechanical thrombectomy for ACIS. The ROC analyses suggest that the iMCA SBF and preoperative NIHSS score might predict good outcomes by 90 days after the intervention. While further confirmation using larger studies is mandatory, these findings suggest that TCD might have the potential to play a larger future role in the prognostic assessment of ischemic stroke patients.

Imaging modalities like magnetic resonance angiography, CTA, DSA, arterial spin labeling, single-photon emission CT, and positron emission tomography all play a role in ischemic stroke management [20–23]. However, TCD sonography provides unique real-time insights into the hemodynamic findings and can contribute to hemodynamics stabilization during patient care without the use of ionizing radiation or invasive procedures. TCD is widely available and very affordable, but TCD is highly dependent upon the skill level and experience of the operator, and its diagnostic and prognostic ability has been debated in patients with sufficient acoustic windows [20,24,25]. TCD can also be used to guide patient management to normalize hemodynamics. Low et al used TCD to monitor the hemodynamics in patients with severe stenoocclusive disease of the ICA or MCA treated by STA-MCA bypass surgery or medical treatment [26]. When hyperperfusion (SBF 50% greater than the pre-procedure value) occurs, blood flow can be decreased by lowering blood pressure [27]. When the MBF velocity in the MCA decreases by more than 50%, short-term balloon occlusion can be indicated [28]. TCD sonography can provide hemodynamic insights as part of the diagnostic armamentarium and, as highlighted in this study, might be a useful prognostic indicator. Of note, it was also independent of the preoperative NIHSS, ICH after therapy, and ASPECTS.

Multiple studies have reported that hemodynamic findings apparent on TCD after endovascular interventions for stroke have diagnostic and prognostic value. Kneihsl et al. [29] found that TCD sonography showed a high MCA MBF velocity index (MBF velocity of the recanalized MCA divided by the cMCA) after 24 h of successful recanalization for ACIS was predictive of ICH and poorer functional outcomes [30]. These findings are likely related to hyperperfusion syndrome or reperfusion injury, which is difficult to detect *via* conventional imaging and can occur regardless of individual differences and compensatory circulatory changes. Another study confirmed that, in addition to MCA, a mean velocity of 30% greater in the iACA versus the contralateral artery within seven days of endovascular intervention was correlated to good functional outcomes in patients with acute MCA/ICA occlusive stroke [31]. Taking the individual differences into account, however, these studies do not fully address the possibility of compensatory change or high perfusion of ACA. Thus, this study contributes a meaningful additional sample to a growing body of literature suggesting the prognostic power of TCD in ACIS.

In this study, cases with ipsilateral stenosis of MCA, ACA, and ICA were excluded; on the other hand, TCD examination was completed 10–14 days after the procedure, which attenuated the effect of post-procedural hyperperfusion that occurs 12 h after endovascular treatment and generally peaking at 1.7 ± 2.1 days [32]. Therefore, the blood flow velocity of the MCA is a more realistic response to ipsilateral cerebral perfusion and the post-procedure state of cerebrovascular function [33]. These findings are consistent with prior studies. Wu et al. [31] showed that the presence of flow diversion at seven days (ipsilateral flow velocity 30% higher than the contralateral) after stroke was associated with good outcomes. Baracchini et al. [34] reported that TCD sonography was a useful prognostic indicator in anterior circulation stroke.

The NIHSS score is a well-known prognostic score for stroke. This study predicted good outcomes after mechanical thrombectomy for ACIS. These results are supported by the literature [35–38], which provides some external validity to this study. Furthermore, the association of the preoperative NIHSS score with good outcomes was independent of iMCA SBF, ICH after therapy, and ASPECTS.

The limitations of this study include a single-center design and a relatively small sample size, which might limit the detectability of prognostic signals and differences between demographic groups. Furthermore, baseline TCD data before procedures were not available, necessitating future prospective studies to confirm changes between baseline and post-surgical hemodynamics of different individuals. Only the occurrence of ICH could be analyzed, not its duration or its severity. It was also notable that hyperperfusion was generally observed within 24 h of endovascular intervention in this study, though literature reports in the general population suggest that hyperperfusion can occur up to day 12 following mechanical thrombectomy [29]. Finally, the follow-up period was short. While further prospective confirmation is needed, these preliminary findings might suggest the prognostic value of TCD sonography, consistent with the literature.

In conclusion, TCD might be a tool to monitor the functional outcomes in patients undergoing mechanical thrombectomy for ACIS. TCD iMCA SBF and preoperative NIHSS scores might be predictive of good outcomes. Additional large, prospective studies are mandatory to validate the predictive role of hemodynamic findings the following recanalization for anterior circulation stroke and to support the clinical use of TCD as a prognostic indicator.

## Abbreviations

ACA: anterior cerebral artery
ASL: arterial spin labeling
CT: computed tomography
CTA: computed tomography angiography
DBF: diastolic blood flow
DSA: digital subtraction angiography
ICA: internal carotid artery
ICH: intracranial hemorrhage
ICU: intensive care unit
MBF: mean blood flow
MCA: middle cerebral artery
MRA: magnetic resonance angiography
mRS: modified Rankin scale
NIHSS: national institutes of health stroke scale
PCA: posterior cerebral artery
PET: positron emission tomography
PI: pulsatility index
RI: resistance index
ROC: receiver operating characteristic
r-tPA: recombinant tissue plasminogen activator
SBF: systolic blood flow
SPECT: single-photon emission CT
TCD: transcranial Doppler ultrasound
TICI: thrombolysis in cerebral infarction
